# Low Tidal Volume Ventilation in Percutaneous Liver Ablations: Preliminary Experience on 10 Patients

**DOI:** 10.3390/diagnostics15121495

**Published:** 2025-06-12

**Authors:** Francesco Giurazza, Francesco Coletta, Antonio Tomasello, Fabio Corvino, Silvio Canciello, Claudio Carrubba, Vincenzo Schettini, Francesca Schettino, Romolo Villani, Raffaella Niola

**Affiliations:** 1 Vascular and Interventional Radiology Department, Cardarelli Hospital, Via Antonio Cardarelli 9, 80131 Naples, Italy; fabio.corvino@aocardarelli.it (F.C.); claudio.carrubba@aocardarelli.it (C.C.); raffaella.niola@aocardarelli.it (R.N.); 2 Emergency and Acceptance Department, Anaesthesia, Emergency and Burn Intensive Care Unit and Poison Control Center, Cardarelli Hospital, Via Antonio Cardarelli 9, 80131 Naples, Italy; francesco.coletta@aocardarelli.it (F.C.); antonio.tomasello@aocardarelli.it (A.T.); silvio.canciello@aocardarelli.it (S.C.); vincenzo.schettini@aocardarelli.it (V.S.); francesca.schettino@aocardarelli.it (F.S.); romolo.villani@aocardarelli.it (R.V.)

**Keywords:** low tidal volume ventilation, thermal ablation, liver, anesthesiological, microwaves, percutaneous

## Abstract

**Objectives:** Low tidal volume ventilation (LTVV) is a ventilatory strategy with the advantages of minimizing diaphragm movements and reducing hypercapnia and barotrauma risks. This preliminary study aims to report on the safety and effectiveness of LTVV applied during percutaneous US-guided liver ablations of focal malignancies. **Methods:** Patients affected by focal liver malignancies treated with percutaneous microwaves ablation were retrospectively included in this single-center analysis. Arterial gas analysis was performed immediately before and after ablation to evaluate the arterial pH, partial pressure of carbon dioxide (pCO_2_), partial pressure of oxygen (pO_2_), and plasma lactate levels. The primary endpoint of this study was to evaluate the safety and efficacy of LTVV during percutaneous liver cancer ablation. The secondary endpoint was to assess the procedural technical success in terms of correct needle probe targeting without the need for repositioning. **Results:** Ten patients affected by a single liver lesion had been analyzed. The ASA score was three in all patients, with three patients also suffering from COPD. The procedural technical success was 100%: ablations were performed with a single liver puncture without the need for changing access or repositioning the needle. No variations in post-ablation arterial gas analysis requiring anesthesiological management remodulation occurred. Lactate levels remained stable and hemodynamic balance was preserved during all procedures. No switch to standard volume ventilation was required. **Conclusions:** In this preliminary study, LTVV was a safe and effective anesthesiological protocol in patients treated with percutaneous ablations of liver malignancies, offering an ideal balance between patient safety and percutaneous needle probe positioning precision. Larger prospective studies are needed to confirm these findings.

## 1. Introduction

Percutaneous thermal ablations are established interventions used with curative intent to treat focal liver neoplasms, both primary and secondary [1], and have been included in multiple international guidelines [2,3,4,5].

These procedures allow for the destruction of a liver lesion by positioning a needle probe through the skin directly into the target under image guidance: ultrasound (US), computed tomography (CT), and magnetic resonance (MR) can be applied, but US has been the most commonly adopted thanks to its real-time control, wide availability, and cost-effectiveness [1].

Ablation success depends on the precise positioning of the needle probe into the target, while at the same time allowing for cancer necrosis and healthy liver preservation; apart from an operator’s technical skills, anesthesiological management is crucial because these procedures are painful and consequently deep sedation is mandatory. However, respiratory excursions induce wide liver movements that could provoke needle probe displacement, causing reduced technical precision, off-target ablations, and damage to adjacent structures [6,7]; during a normal respiratory cycle, the liver moves with an amplitude of 1–2 cm [8].

Therefore, different anesthesiological protocols have been proposed, including conscious sedation, standard mechanical ventilation, and high-flow jet ventilation (HFJV), but a standard approach has still not been identified, with each protocol presenting advantages and drawbacks [6,7,9,10,11].

During conscious sedation, a patient’s respiratory movements may limit precision and procedural success because spontaneous breathing under conscious sedation does not allow for the control of thoracic and visceral motion [12].

Standard mechanical ventilation under general anesthesia may have a lower physiological impact on the patient but also introduces predictable thoracic excursions, which can significantly interfere with interventional accuracy [13].

HFJV has been recently introduced in thermal ablations [14], presenting the advantage of minimizing diaphragm excursion and enhancing proper needle positioning in a motionless liver target; it consists of mechanical ventilation during general anesthesia by delivering high-flow and short-duration pulses of pressurized gas in the trachea via a small caliber catheter, where expiration is passive; however, this presents limitations in patients with coexisting chronic obstructive pulmonary disease (COPD) due to a potential risk of hypercapnia and barotrauma [11,15].

Furthermore, patients undergoing liver ablation may present with comorbidities [1,2] in deteriorating clinical conditions, so the choice of anesthesiological strategy remains challenging and it is still a topic of ongoing debate.

On the other hand, low tidal volume ventilation (LTVV) is a ventilatory strategy primarily used in mechanically ventilated patients, especially those with acute lung injury or acute respiratory distress syndrome [16]; its advantages are reductions in diaphragm and hepatic movements with at the same time minimal risk of hypercapnia and barotrauma; until now, few studies have been available concerning its application in atrial ablation interventions [17,18,19], which have exhibited promising results both in terms of technical and clinical success rates; however, no data regarding applications of LTVV in abdominal organ ablations have been published.

This study aims to report on a preliminary experience concerning the safety and effectiveness of an LTVV protocol applied during percutaneous US-guided liver ablations of focal malignancies.

## 2. Materials and Methods

This is a monocentric retrospective study analyzing patients treated with percutaneous US-guided thermal ablation of focal liver lesions under LTVV ventilation; patients were treated during the second semester of 2024.

Local ethical committee approval was obtained (DEL: 233/2024); written informed consent was acquired from all patients before the intervention.

### 2.1. Study Protocol

Patients affected by focal liver malignancies, both primary and secondary, undergoing thermal ablation were included in this analysis.

All cases had been previously discussed in a multidisciplinary liver tumor board.

The inclusion criteria were patients undergoing percutaneous liver ablation with LTVV (defined as tidal volumes less than 6–8 mL/kg of the predicted body weight), the availability of arterial blood gas analysis, and preserved hepatic, renal, and coagulation functions. Exclusion criteria included acute respiratory disease, COPD classified as GOLD stage III or higher, the need for orotracheal intubation, anatomical abnormalities incompatible with laryngeal mask airway, additional liver interventions during the same session (endovascular embolizations), the presence of ascites, and concomitant infectious diseases.

A contrast-enhanced CT or MR scan was acquired no more than 5 weeks before the treatment to plan the intervention.

The preprocedural assessment included standard laboratory exams (blood count, coagulation, electrolytes, liver, and renal functions), an ECG evaluation, and an anesthesiological evaluation with the ASA (American Society of Anesthesiologists) score.

Arterial gas analysis was performed immediately before and after the ablation to evaluate the arterial pH, partial pressure of carbon dioxide (pCO_2_), partial pressure of oxygen (pO_2_), and plasma lactate levels.

Patients were monitored clinically and radiologically up to one month after the intervention in order to detect eventual complications related to the ventilation strategy and verify short-term procedural outcomes.

The primary endpoint of this study was to evaluate the safety and efficacy of LTVV during percutaneous liver cancer ablation; the secondary endpoint was to assess the procedural technical success in terms of correct needle probe targeting without the need for repositioning.

### 2.2. Thermal Ablation Intervention

All interventions were performed in a hospital setting in an angio suite under US-guidance (Aplio a—Canon^®^ Medical Systems Corporation, Otawara, Japan).

Interventional radiologists and anesthesiologists had >10 years of experience in thermal ablation procedures; overall, three radiologists and five anesthesiologists were involved, following a standard protocol in order to avoid interoperator variation.

As appropriate, antiplatelet and anticoagulation drugs were managed according to international guidelines [20,21], as well as considering to any anomalies in the coagulation values (platelet count and INR).

Antibiotic prophylaxis (2gr Cefazoline) was given intraprocedurally.

The liver target was examined during US in both plane- and contrast-enhanced scans (Sonovue—Bracco^®^, Milan, Italy).

Intercostal access was selected to reduce bleeding complications; in the case of visceral structures (gallbladder, stomach, and colon hepatic flexure) located close to the target, percutaneous needle-guided hydrodissection with dextrose was conducted before positioning the needle probe.

The need to reposition the needle probe and/or change intercostal space access were recorded to ensure technical success.

Ablations were performed with a microwaves needle probe (Emprint—Medtronic^®^, Boulder, CO, USA) applying an energy of 150 W during the amount of time indicated by the manufacturer to achieve the desired area of necrosis; a 5 mm or a 10 mm additional ablation margin was applied in primary and secondary lesions, respectively, to minimize the risk of cancer remnants [1].

Before starting the ablation phase, proper needle probe positioning into the target was verified in multiple US planes and measurements were conducted to assess adequate lesion covering.

**Figure 1:**
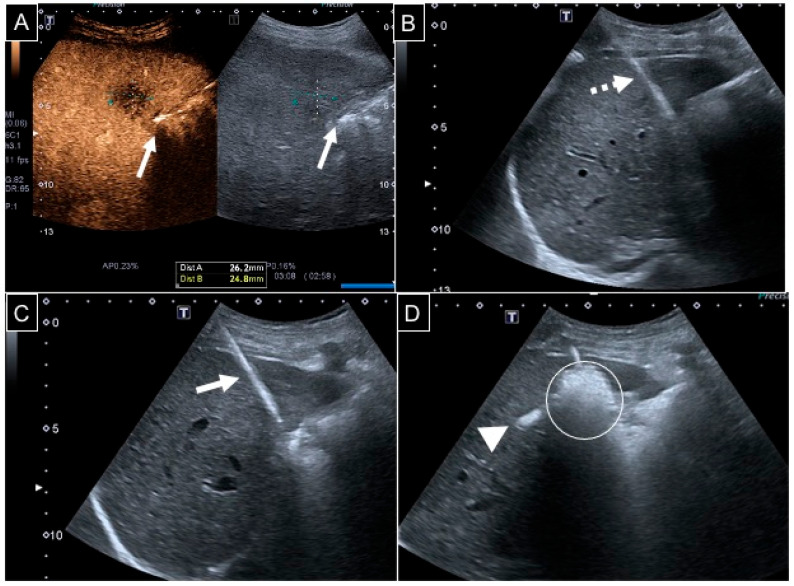
A 67-year-old man with a 26 mm liver metastasis from colon adenocarcinoma in segment V. (**A**) Contrast-enhanced US showing a hypovascularized lesion adjacent to the gallbladder wall (white arrows); (**B**) considering that the ablation area would cover a 10 mm safety margin around the target, hydrodissection with 50 mL of dextrose was performed via a 22 G 10 cm spinal needle (dotted arrow) under US guidance; (**C**) a 13 G needle probe (white arrow) was then positioned with an intercostal access; (**D**) ablation was conducted at 150 W for 3 min: the ablation area appeared hyperechoic (white circle), and a hepatic vein heat steal effect was evident too (white arrowhead).

Ablations were performed under continuous real-time US monitoring (Figure 1); once concluded, the needle probe was retrieved under track ablation to seal the percutaneous tract and avoid cancer seeding.

At the end, a plane CBCT was acquired to detect any eventual acute complications (pneumothorax, visceral perforations, or perihepatic fluid). CIRSE standards for complications grading [22] was applied.

At 1 month from the intervention, a contrast-enhanced CT scan was acquired to verify the outcome of the procedure.

### 2.3. Anesthesiological Management

According to the preoperative anesthesiological assessment, patients were deemed eligible for deep sedation using a supraglottic airway device.

Intraprocedural monitoring included heart rate frequency, oxygen saturation, and non-invasive blood pressure. Peripheral 18 G venous access was positioned on the left hand.

The intraoperative anesthesiological protocol consisted of two distinct phases (Table 1):

- Phase 1 focused on target localization and the proper placement of the ablation needle probe: It was conducted under local anesthesia by the interventional radiologist; 20 mL of mepivacaine 3% were injected via a 22 G spinal needle in subcutaneous tissues and on the Glissonian capsule under US guidance according to the skin entry point selected for subsequent needle probe access. Analgesia using fentanyl (1.5 mcg/kg) and midazolam (0.03 mg/kg) was combined. The patient remained conscious to allow for active participation in controlling diaphragmatic excursions.
- Phase 2 corresponded to the delivery of ablative energy: It was carried out under deep analgesic sedation using continuous intravenous infusion of propofol (1–2.5 mg/kg for induction and 6–12 mg/kg/h for maintenance) and remifentanil (0.1 mcg/kg/min). Ventilation was provided with a conventional ventilator via a laryngeal mask airway i-gel size 4, using controlled mechanical ventilation with low tidal volumes (2.0–2.5 mL/kg), an increased respiratory rate (18–22 breaths per minute), 100% fraction inspiration oxygen (FiO_2_), and a positive end-expiration pressure (PEEP) of 5 cm H_2_O. Thermal ablation was delivered during a variable time based on the needle manufacturer protocol according to the lesion diameter (range: 3–9 min). The sedation level was assessed by applying the Richmond Agitation–Sedation Scale (RASS) intraoperatively and three minutes after removal of the supraglottic device. Arterial gas analysis was performed at two time points: before anesthesiological induction and immediately at the end of the intervention.

After ablation, an elastomeric pump was set up with 20 mg of morphine diluted in 60 mL of 0.9% NaCl at a rate of 2 mL/h, with rescue analgesia consisting of 1 gr of paracetamol every 12 h.

LTVV safety was defined by the following criteria: no clinically significant changes in post-ablation arterial blood gas parameters (pH, pCO_2_, pO_2_, and lactates) that would require anesthesiological management adjustments; stable post-procedure hemodynamic balance, not requiring vasopressors or fluid resuscitation; adequate sedation level at awakening assessed by the Richmond Agitation–Sedation Scale (RASS), with a strict target of ≥−1. This RASS threshold was adopted to ensure that patients were neither overly sedated nor agitated upon awakening, providing a clear, standardized measure of sedation adequacy as part of the safety assessment.

LTVV effectiveness was defined as completed ablation procedures under LTVV without the need to switch to standard mechanical ventilation.

### 2.4. Statistical Analysis

Descriptive analysis included continuous variables expressed as mean ± standard deviation and categorical variables expressed as frequencies and percentages.

All data analysis was performed in SPSS v.29 (IBM^®^, Armonk, NY, USA).

## 3. Results

Ten patients (six men and four women; mean age: 65.9 years; age range: 51–83 years) have been analyzed in this study.

Three had primary cancers (hepatocarcinoma), while seven were affected by liver metastasis. Each patient had a single hepatic lesion.

The mean lesion diameter was 26 mm (range: 16–41 mm); lesions were located in segments 4b (1), 5 (1), 6 (2), 7 (3), and 8 (3) (Table 2).

Hydrodissection with dextrose was conducted in three cases (two for colonic hepatic flexure and one for gallbladder).

**Figure 2:**
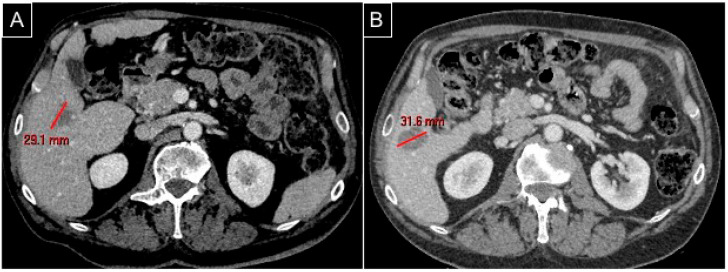
The same patient as Figure 1. (**A**) A preprocedural CT scan in the venous phase axial plane detecting the hypovascularized metastasis in segment V; (**B**) a postprocedural CT scan in the venous phase axial plane for follow-up at 2 months showing complete ablation with an ovoid regular margin without remnant.

At the final CBCT, perihepatic fluid was detected in only one patient and without clinical sequelae; at the CT scan follow-up, no lesion remnant or relapse was detected (Figure 2).

Procedural technical success was 100%: ablations were performed with a single liver puncture without the need for changing intercostal access or repositioning during the ablation phase.

Two grade I complications occurred (abdominal pain), requiring additional painkillers without prolonged hospitalization; one grade II complication was detected (liver abscess) and was managed conservatively. No major complications were recorded.

### Arterial Gas Analysis Outcomes

The ASA score was three in all patients; three patients suffered from COPD.

The mean preprocedural pH value was 7.38 ± 0.02, while the mean postprocedural pH was reduced (7.37 ± 0.02); this slight shift toward respiratory acidosis was characterized by a global increase in pCO_2_. Indeed, the mean preprocedural pCO_2_ was 42.4 ± 4.5 mmHg and the mean postprocedural pCO_2_ was 46.2 ± 5.1 mmHg (Table 3).

Six patients had a postprocedural increase in pCO_2_: the maximum value increase was from 48 to 54 mmHg.

The mean preprocedural and postprocedural levels of pO_2_ were 91.2 ± 3.2 mmHg and 133.0 ± 14.3 mmHg, respectively.

Lactate levels remained approximately stable: the mean preprocedural value was 0.92 mmol/L (range: 0.6–1.7 mmol/L), while the mean postprocedural value was 1.13 mmol/L (range: 0.7–1.4 mmol/L).

The hemodynamic balance remained stable during all interventions.

Regarding the RASS scores, all patients had an intraprocedural value included between −3 and −4. At 3 min from removal of laryngeal mask, the mean RASS score was 0.2: seven patients presented with 0 and three patients presented with −1.

Hence, the LTVV safety rate was 100%.

There were no significant appreciable differences in the arterial gas analysis values when comparing patients with and without COPD, even if a slight increase in pCO_2_ was more frequently observed in those with COPD (mean values: 6.0 ± 1.4 mmHg vs. 3.4 ± 1.2 mmHg, *p* = 0.07).

No switch to standard volume ventilation was required, with 100% LTVV effectiveness.

## 4. Discussion

In this study, according to the authors’ knowledge, LTVV was for the first time reported as the anesthesiological protocol adopted during percutaneous liver ablation procedures; the results show that it is safe and effective. Procedural success was achieved in all cases, which also suggests easier needle probe targeting and control for the interventional radiologist due to reduced liver movements; this aspect is of paramount importance during any percutaneous liver ablation intervention because diaphragmatic excursions can hinder lesion targeting and also induce needle dislodgement from its correct location, causing off-target ablation areas and damage to healthy structures [7].

These findings indicate that LTVV application during image-guided ablation would provide advantages for both anesthesiologists and interventional radiologists, improving patient tolerance and ablation precision.

Previous studies have already demonstrated that a reduction in liver excursions during a liver ablation procedure led to improved accuracy, safety, and success [23]. Without liver movements, the needle probe trajectory from the skin entrance to the target can be precisely planned even in unfavorable locations, such as under diaphragmatic lesions, with improved clinical results.

Compared to LTVV, conventional mechanical ventilation creates breathing-related motions that can lead to imprecision of the probe placement; therefore, alternative strategies have been investigated and until now the most adopted protocol to reduce diaphragm motion during liver ablation has been HFJV [7,9,10,11,24,25].

Different authors have reported the usefulness of HFJV during percutaneous ablations: Denys et al. [9] demonstrated a decrease in organ movement from 20 to less than 1 mm during liver, kidney, and lung tumor ablations; Abderhalden et al. [7] treated 19 patients affected by liver or renal tumors under CT guidance without complications and with less radiation exposure; similar findings were reported by Chung et al. [25] in CT-guided lung ablations. Recently, Trochu et al. [11] reported on 60 patients treated with percutaneous ablation of renal or liver cancers using HFJV without respiratory complications, low gas exchange modifications and minimal lung volumes changes; interestingly, in their series, 40% of patients were obese, 57% had severe systemic disease (ASA scores of three), and 12% had chronic respiratory diseases. However, COPD, recent pneumothorax, and severe obesity remain contraindications to HFJV [15], according to findings coming from ear, nose, and throat interventions, but these limitations may differ in abdominal ablation interventions.

In our study with LTVV, all patients had severe comorbidities with ASA scores of three and three subjects also had COPD. The slight increase in pCO_2_ recorded after the intervention was associated with a minimal pH reduction, not requiring conversion to standard mechanical ventilation and indicating a well-tolerated hypercapnia as an acceptable collateral effect [26]. Regarding oxygenation, an increase in pO_2_ values (from 91.2 ± 3.2 to 133.0 ± 14.3 mmHg) was appreciated, relating to an optimized ventilation/perfusion ratio and adequate PEEP levels during ablation; at the same time, lactate values remained stable, indicating an adequate tissue perfusion without systemic hypoxia nor metabolic stress. Hemodynamic balance was preserved during all procedures, confirming LTVV anesthesiological safety, even in those patients with COPD, without additional pharmacological support. Sedation monitoring via RASS suggested an optimal sedation and a fast neurological recovery. In our study, LTVV was given via laryngeal masks, which are especially useful in those interventions where a minimal visceral impact is required; our findings are in line with previous publications on atrial fibrillation ablations, where LTVV were already proven to be safe and effective in airway management [17,27,28,29], while also improving catheter stability and lesion durability compared to traditional ventilation methods [30,31].

So, while conventional mechanical ventilation creates breathing-related motions leading to probe placement imprecision, HFJV overcomes this issue but entails a potential risk of hypercapnia and barotrauma, especially in patients affected by COPD; LTVV could combine the advantages of HFJV in terms of liver motionless but with the safety of conventional mechanical ventilation regarding gas exchange and barotrauma occurrence.

Although no significant differences were observed between patients with and without COPD in terms of arterial blood gas analysis, in our study a slight trend toward increased pCO_2_ levels in COPD patients was detected. This finding highlights the need for a more detailed subgroup analysis to understand how differently LTVV may affect respiratory function and tolerance among populations with varying pulmonary comorbidities.

This study presents preliminary results and so it has limitations: first of all, the number of patients is small with a monocentric retrospective design, so data on larger multicentric sample are required, especially focusing on those patients affected by COPD who would be the ones benefiting more from LTVV; also, no control group with a standard mechanical ventilation or HFJV was available and so a direct comparison cannot be achieved; only US-guided liver ablations have been considered and studies also including other image guidance strategies (CT, MR, and CBCT) and additional visceral targets (kidney, adrenal glands, lungs, etc.) should be conducted. Finally, only patients with a single lesion were included, so the applicability of LTVV should also be assessed in cases involving multiple ablation sites within the same session. Future studies incorporating larger, diverse populations, extended follow-up, and appropriate control groups will be essential to validate and expand upon these findings.

## 5. Conclusions

In this preliminary study, LTVV was a safe and effective anesthesiological strategy in patients treated with percutaneous ablations of liver cancer.

LTVV reduced liver movements related to diaphragmatic excursions, offering an ideal balance between patient safety and percutaneous needle probe positioning precision.

Future prospective studies with larger samples are required to validate these data.

## Figures and Tables

**Table 1:** Anesthetic protocol.

Phase 1: Needle Probe Targeting	Phase 2: Ablation
Local anesthesia	Deep analgesic sedation
*20 mL mepivacaine 3% subcutaneous and periglissonian*	*1–2.5 mg/kg propofol e.v. for induction*
	*6–12 mg/kg/h propofol e.v. for maintenance*
	*0.1 mcg/kg/min remifentanil e.v.*
Analgesia	
*1.5 mcg/kg fentanyl e.v.*	Ventilation
*0.03 mg/kg midazolam e.v.*	*mechanical LTV 2.0–2.5 mL/kg, RR 18–22 br/min, 100% FiO_2_, PEEP 5 cm H_2_O*

mL: milliliter; mg: milligram; kg: kilogram; e.v.: endovenous; h: hours; mcg: microgram; LTV: low tidal volume; RR: respiratory rate; br: breath; min: minute; FiO_2_: fraction inspiration oxygen; PEEP: positive end-expiration pressure; cm: centimeter.

**Table 2:** Patient population characteristics.

Patient	Sex	Age	Cancer	Size (mm)	Segment	COPD
*1*	F	71	Colon met	20	VII	no
*2*	M	83	Colon met	30	VIII	yes
*3*	M	69	HCC	35	VIII	yes
*4*	F	54	Breast met	16	IVb	no
*5*	F	51	Breast met	19	VI	no
*6*	M	77	HCC	41	VI	yes
*7*	M	57	HCC	23	VII	no
*8*	M	75	Colon met	31	VII	no
*9*	M	67	Colon met	26	V	no
*10*	F	55	Colon met	18	VIII	no

**Table 3:** Arterial gas analysis and the Richmond Agitation–Sedation Scale.

Pt	pH (Pre)	pH (Post)	pCO_2_ (Pre)	pCO_2_ (Post)	pO_2_ (Pre)	pO_2_ (Post)	Lactate (Pre)	Lactate (Post)	RASS (intra)	RASS (3 min)
*1*	7.4	7.38	42	48	90	110	0.6	0.7	−4	0
*2*	7.36	7.38	46	42	88	130	0.8	1	−3	0
*3*	7.4	7.35	48	54	89	130	0.8	1.1	−3	0
*4*	7.37	7.38	39	43	95	150	1.1	1.3	−3	−1
*5*	7.39	7.35	37	42	94	145	0.7	1.1	−3	0
*6*	7.4	7.36	45	50	80	103	1.1	1.3	−4	0
*7*	7.41	7.36	44	52	70	140	1.7	1.4	−3	−1
*8*	7.38	7.35	40	48	80	130	0.8	1.2	−3	0
*9*	7.43	7.36	38	44	88	160	0.9	1.3	−4	0
*10*	7.47	7.37	49	43	90	110	0.7	1	−3	0

Pt: patient; RASS: Richmond Agitation–Sedation Scale.

## Data Availability

The raw data supporting the conclusions of this article will be made available by the authors on request.
